# Association between depressive symptoms and thoracic aortic calcification, and their independent and joint prognostic value for incident atherosclerotic cardiovascular disease in the MESA cohort

**DOI:** 10.1007/s10554-026-03771-0

**Published:** 2026-08-19

**Authors:** Daiki Arai, Quinn White, Keishi Ichikawa, Robyn L. McClelland, W. Craig Johnson, Mio Yabuki, Spencer Hansen, Matthew J. Budoff

**Affiliations:** 1 Lundquist Institute, Harbor-UCLA Medical Center, 1124 W. Carson St, Torrance, CA 90502 USA; 2 Faculty of Medicine, University of Debrecen, Nagyerdei krt. 94., 4032 Debrecen, Hungary; 3 Department of Biostatistics, Hans Rosling Center for Population Health, University of Washington, 3980 15th Avenue NE, Box 351617, Seattle, WA 98195-1617 USA

**Keywords:** Thoracic aortic calcification, Depressive symptoms, ASCVD, Risk stratification, MESA

## Abstract

Depression is highly prevalent; however, its association with atherosclerotic cardiovascular disease (ASCVD) events remains incompletely understood. This study aimed to evaluate the association between depressive symptoms and thoracic aortic calcification (TAC) and assess their joint prognostic value for future ASCVD events. We analyzed 2,600 participants from the Multi-Ethnic Study of Atherosclerosis (MESA) without baseline ASCVD. TAC was quantified using non-contrast chest CT as a marker of subclinical atherosclerosis. Depressive symptoms were assessed using the Center for Epidemiologic Studies Depression Scale (CES-D ≥ 16). Linear regression assessed the association between depressive symptoms and log-transformed TAC. Cox proportional hazards models evaluated independent and joint associations of depressive symptoms and TAC tertiles with incident ASCVD, adjusting for covariates. Overall, 389 participants (15%) exhibited depressive symptoms. In fully adjusted models, depressive symptoms were not associated with TAC (β = 0.08; 95% CI -0.13–0.29), without sex-specific differences. Both depressive symptoms (HR 1.63; 95% CI 1.16–2.29) and TAC (HR 1.22; 95% CI 1.13–1.33) were independently associated with ASCVD events. Individuals with depressive symptoms had significantly higher ASCVD risk in the highest tertile TAC group, whereas no difference was observed in lower TAC tertiles. Although depressive symptoms were not directly associated with TAC, their presence was significantly associated with increased ASCVD risk, particularly among participants with elevated TAC. Coexisting depressive symptoms and substantial atherosclerotic burden confer a higher ASCVD risk. Thus, evaluating depressive symptoms may provide additional context for cardiovascular risk stratification, particularly among individuals with advanced subclinical atherosclerosis.

**Supplementary Information:**

The online version contains supplementary material available at https://doi.org/10.1007/s10554-026-03771-0.

## Introduction

Cardiovascular disease remains the leading cause of mortality worldwide, accounting for nearly one-third of all deaths in the United States, underscoring the importance of atherosclerotic cardiovascular disease (ASCVD) prevention and risk stratification [1]. Depression is also highly prevalent in the general population, affecting 8.3% of all U.S. adults in 2021, with an even higher prevalence among patients with cardiovascular diseases (20.8%) [2, 3]. Cardiovascular disorders predispose individuals to depression through multiple mechanisms, including symptom burden, psychological stress, financial hardships, and functional limitations [4]. Conversely, the presence of depression is associated with an increased risk of developing ASCVD [5]. Despite this bidirectional relationship, the biological mechanisms linking depression to heightened cardiovascular risk remain incompletely understood [3].

Atherosclerosis is the primary underlying pathological process driving cardiovascular diseases [6]. Imaging modalities are widely used to detect and quantify the extent of atherosclerosis [7]. Thoracic aortic calcification (TAC), detected and quantified by non-contrast chest CT scans, serves as a robust marker of the total burden of aortic atherosclerosis and a validated predictor of future ASCVD events [8, 9]. Several prior studies have evaluated the association between depressive symptoms and aortic calcification extending from the aortic arch to the iliac bifurcation using electron beam tomography (EBT) [10–12]. Although these studies found that a history of recurrent major depression was associated with a significant increase in the risk of aortic calcification, aortic calcification in the existing data were assessed without the ascending aorta. In addition, participants enrolled in these studies were exclusively female and predominantly Caucasian. Thus, no prior study has examined the association between depressive symptoms and TAC across the ascending aorta, aortic arch, and descending thoracic aorta using chest CT in a large, multiethnic population.

MESA is a large, ethnically diverse cohort study that provides standardized chest CT scans with quantified TAC scores, along with depressive symptom assessment based on the Center for Epidemiologic Studies Depression Scale (CES-D) and long-term follow-up for cardiovascular outcomes. Therefore, utilizing the MESA cohort, this study aims to examine the association between depressive symptoms and atherosclerosis measured by TAC, including potential sex-specific differences, and to evaluate the joint association of depressive symptoms and TAC with future ASCVD events.

## Materials and methods

### Study population

**Fig. 1:**
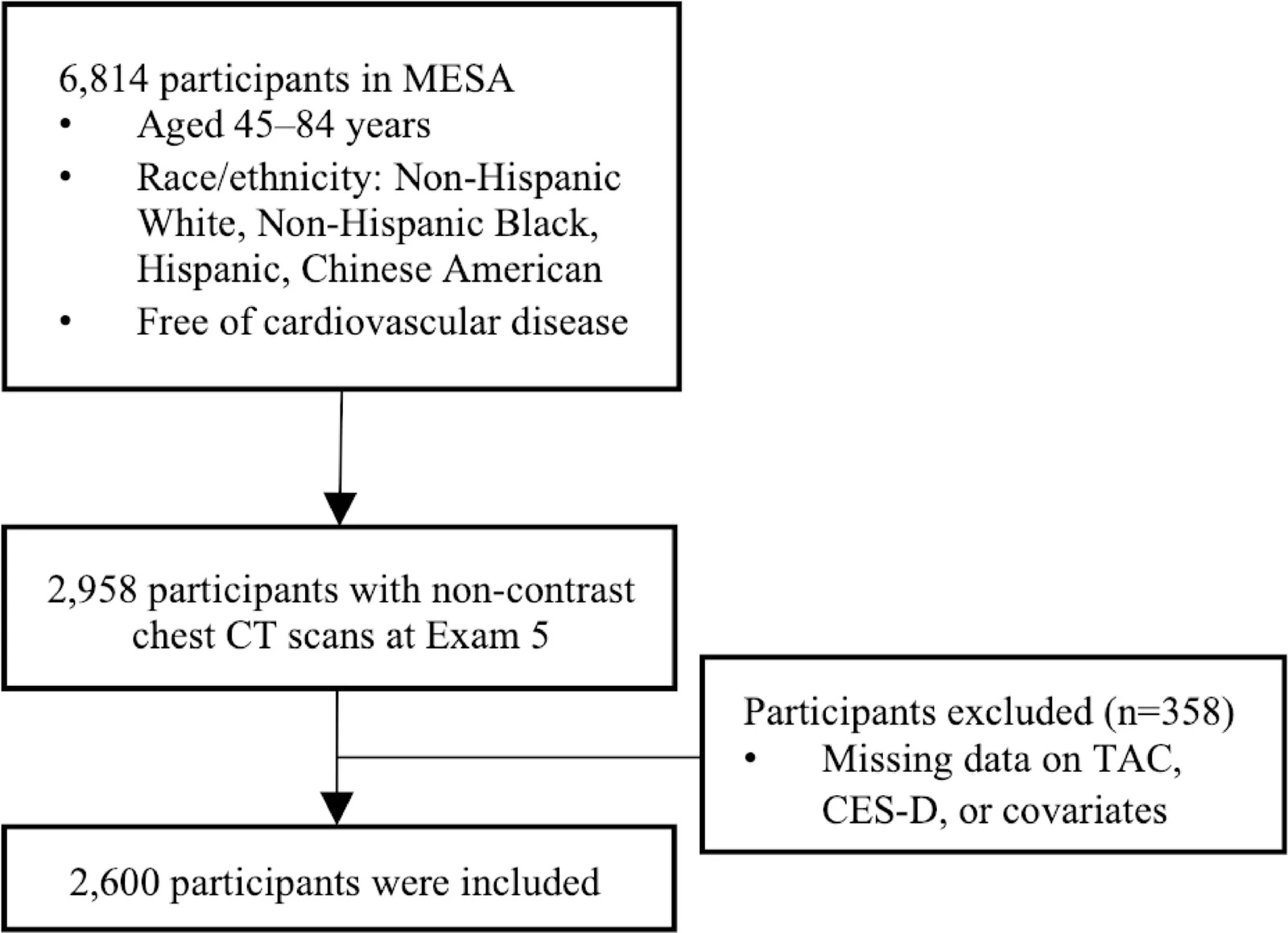
Participant Selection Flow. Flow diagram illustrating participant selection from the MESA. Of 6,814 participants enrolled at baseline and free of cardiovascular disease, 2,958 underwent non-contrast chest CT at Exam 5. After exclusion of participants with missing TAC, depressive symptom assessed by the CES-D, or covariate data (*n* = 358), a total of 2,600 participants were included in the analysis. CES-D, Center for Epidemiologic Studies Depression Scale; MESA, Multi-Ethnic Study of Atherosclerosis; TAC, thoracic aortic calcification

MESA is a prospective cohort study designed to investigate the prevalence and progression of subclinical ASCVD, and to identify risk factors for incident ASCVD in a racially and ethnically diverse community-based sample. From July 2000 through August 2002, 6,814 men and women aged 45 to 84 years and free of clinical cardiovascular disease were recruited from six different sites in the United States (Baltimore, Maryland; Chicago, Illinois; Forsyth County, North Carolina; Los Angeles, California; New York, New York; and St. Paul, Minnesota). The study design of MESA has been previously published [13]. This study included all the participants who underwent non-contrast chest CT scans in MESA Exam 5 (April 2010 to December 2011). Figure 1 shows a flow diagram illustrating participant selection in this study. A total of 2,958 participants had non-contrast chest CT. Of these, 358 participants were excluded due to missing data. After these exclusions, 2,600 participants were included in this study. In the subsequent analysis with follow-up data, we excluded 71 participants who had experienced ASCVD events prior to MESA Exam 5. All study participants provided written informed consent. The study was approved by the Institutional Review Board at each field center and the data coordinating center.

### Assessment of TAC score

Non-contrast chest CT scans were acquired at full inspiration following a standardized protocol with reconstruction in 0.625–0.75 mm slice thickness. A detailed description of the TAC score measurement protocol has been published [14]. In brief, TAC was defined as calcification on chest CT in the ascending aorta, aortic arch, and descending thoracic aorta inferior to the cardiac apex. All images were analyzed in a blinded fashion by trained technologists at the CT Reading Center (Lundquist Research Institute, Torrance, California), the same group that has analyzed all CT findings since the inception of MESA. The TAC score was quantified by the Agatston method using the Philips Heart Beat CS software program (Philips Company, Cleveland, Ohio). Intra-reader and inter-reader reproducibility for the TAC score were very good at 99% and 97%, respectively. TAC scores were calculated as the sum of the calcification scores of the ascending aorta, aortic arch, and descending thoracic aorta.

### Assessment of depressive symptoms

Depressive symptoms were assessed using the CES-D questionnaire administered at Exam 5. This instrument is a 20-item self-report questionnaire covering self-report of depressed mood, feelings of worthlessness, feelings of hopelessness, poor concentration, loss of appetite, and sleep disturbance [15]. In the MESA study population, the CES-D was administered in English, Spanish, Cantonese, and Mandarin [13]. Higher scores for the CES-D suggest more evidence of depressive symptoms. In this study, we defined the presence of depressive symptoms to be the CES-D score ≥ 16, a threshold initially reported in Radloff (1977), regardless of the use of antidepressants [16].

### Assessment of covariates

Relevant covariates were obtained from the MESA Exam 5 questionnaire and Exam 5 physiologic assessments. Covariates in this analysis included age, sex, race and ethnicity, education, smoking (pack-years), body mass index (BMI), systolic blood pressure, antihypertensive medication use, lipid-lowering medication use, total cholesterol, high-density lipoprotein (HDL) cholesterol, prevalent diabetes, health insurance status, and known antidepressant use. Interview‐administered questionnaires were used to obtain sociodemographic information, smoking, medication use, and health insurance status. Education was categorized as less than high school, high school graduate, some college, or college graduate and above. Pack-years of cigarette smoking were calculated from age of starting to quitting (or current age among current smokers) × (cigarettes per day/20). Moreover, trained staff collected height, weight, blood pressure, and fasting blood measures. BMI was calculated as weight (kilograms) divided by height (meters) squared. Blood pressure was measured 3 times in the seated position using a Dinamap model Pro 100 automated oscillometric sphygmomanometer (Critikon, Tampa, Florida), and the final 2 measurements were averaged. Total cholesterol and HDL cholesterol levels from blood samples obtained after a 12-hour fast were measured at the Collaborative Studies Clinical Laboratory (Fairview University Medical Center, Minneapolis, Minnesota). Diabetes was defined as fasting blood glucose concentration ≥ 126 mg/dL, self‐reported physician diagnosis, or use of hypoglycemic drugs.

### Outcome data

Participants were regularly contacted via follow-up calls through 2021 to inquire about interim hospital admissions, cardiovascular outpatient diagnoses, and deaths. To verify self-reported diagnoses, information was collected from death certificates and medical records for all hospitalizations and outpatient cardiovascular diagnoses. Detailed description of follow-up of MESA participants is available online (www.mesa-nhlbi.org). ASCVD events were defined as: definite or probable myocardial infarction, resuscitated cardiac arrest, fatal or non-fatal stroke (not transient ischemic attack), coronary heart disease death, and stroke death. A detailed description of the adjudication process has been published [13].

### Statistical analysis

Continuous variables were summarized using the median and interquartile range, while dichotomous variables were reported as counts and proportions. Pairwise comparisons of continuous variables by the presence of depressive symptoms were tested using the Mann–Whitney U-test. Categorical data were compared by the presence of depressive symptoms using chi-squared tests. Linear regression analyses, both adjusted and unadjusted, were conducted to examine the association between depressive symptoms and log-transformed TAC scores. Fully adjusted models included age, sex, race/ethnicity, BMI, systolic blood pressure, pack-years smoking, diabetes, total cholesterol, HDL cholesterol, antihypertensive medication use, lipid-lowering medication use, known antidepressant use, health insurance coverage (yes/no), and education. The association between depressive symptoms and TAC with the hazard of an ASCVD event was evaluated using a fully adjusted Cox proportional hazards model. For this study, time ‘at-risk’ began at the time of the Exam 5 CT scan, and participants who had experienced ASCVD events before that time were excluded. To see how sensitive our results were to our choice of time scale, we included supplementary analyses with an age time scale and handled left truncation by using a counting-process formulation of the event-free survival time with age at Exam 5 chest CT as the entry time and age at the event or censoring as the exit time. Lastly, for ease of interpretation and to allow for nonlinearity in the way the association between CES-D and the hazard of an ASCVD event may vary by TAC, we considered a Cox proportional hazards model comparing each of the six possible combinations of TAC tertile and CES-D group (CES-D ≥ 16 or CES-D < 16), adjusting for the aforementioned set of covariates. Additionally, to visualize the adjusted ASCVD-free survival probabilities across these six groups, inverse probability of treatment weighting (IPTW) was applied to construct adjusted Kaplan-Meier curves.

## Results

### Participant characteristics

Table 1 displays the clinical and laboratory baseline characteristics of the study participants (*N* = 2,600; mean age 68 years; 47% male). At baseline, 15% of the population exhibited depressive symptoms, defined as a CES-D score ≥ 16. The cohort was racially diverse, consisting of 39% White, 27% Black, 20% Hispanic/Latino, and 14% Chinese American participants. Participants were categorized into tertiles based on their baseline TAC scores to facilitate joint association analysis.

**Table 1:** Baseline Clinical and Laboratory Characteristics by Depressive Symptoms

Characteristic	Overall *N* = 2,600	CES-D < 16 *N* = 2,211	CES-D ≥ 16 *N* = 389	*P*-value
Race/Ethnicity				0.14
White	1,024 (39%)	869 (39%)	155 (40%)	
Black	696 (27%)	603 (27%)	93 (24%)	
Hispanic/Latino	528 (20%)	434 (20%)	94 (24%)	
Chinese	352 (14%)	305 (14%)	47 (12%)	
Age	68 (61, 76)	69 (61, 76)	66 (60, 74)	< 0.001
Sex (Male)	1,210 (47%)	1,036 (47%)	174 (45%)	0.5
BMI (kg/m^2)	27.5 (24.5, 31.4)	27.5 (24.4, 31.4)	27.7 (24.9, 32.1)	0.077
Systolic Blood Pressure (mm Hg)	120 (110, 137)	121 (110, 137)	119 (108, 137)	0.2
Pack Years Smoking	0 (0, 16)	0 (0, 15)	2 (0, 20)	0.042
Diabetes	501 (19%)	420 (19%)	81 (21%)	0.4
Total Cholesterol (mg/dl)	181 (158, 207)	182 (158, 208)	180 (156, 205)	0.3
HDL Cholesterol (mg/dl)	53 (44, 64)	53 (45, 64)	52 (43, 63)	0.15
Hypertension Medication Use	1,431 (55%)	1,229 (56%)	202 (52%)	0.2
Lipid-Lowering Medication Use	1,005 (39%)	871 (39%)	134 (34%)	0.073
No Health Insurance	203 (7.8%)	161 (7.3%)	42 (11%)	0.023
Education				0.2
Less Than High School	339 (13%)	276 (12%)	63 (16%)	
High School Graduate	1,218 (47%)	1,044 (47%)	174 (45%)	
College	499 (19%)	428 (19%)	71 (18%)	
Graduate School	544 (21%)	463 (21%)	81 (21%)	
Known Antidepressant Use	269 (10%)	187 (8.5%)	82 (21%)	< 0.001
TAC	285 (46, 1,116)	294 (48, 1,151)	262 (39, 960)	0.2

CES-D, Center for Epidemiologic Studies Depression Scale; HDL, high-density lipoprotein; TAC, thoracic artery calcification

Categorical variables are reported as n (%), and continuous variables are reported as median (Q1, Q3). For comparisons by CES-D group, chi-square tests were performed for categorical variables and Mann-Whitney tests were performed for continuous variables

Participants with depressive symptoms (CES-D ≥ 16) were slightly younger (median 66 vs. 69 years, *p* < 0.001) and had more smoking exposure (median pack-years 2 vs. 0, *p* = 0.042), compared to those without depressive symptoms (CES-D < 16). In addition, the lack of health insurance and known antidepressant use were reported significantly more among those with depressive symptoms (11% vs. 7.3%, *p* = 0.023 and 21% vs. 8.5%, *p* < 0.001, respectively). No significant differences were observed between groups with respect to race/ethnicity, sex, BMI, systolic blood pressure, diabetes, total cholesterol, HDL cholesterol, hypertension medication use, lipid-lowering medication use, and TAC scores.

### Association of depressive symptoms and TAC scores

A linear regression model showed no significant association between depressive symptoms (CES-D ≥ 16) and TAC scores with a coefficient of β = 0.08 (95% CI -0.13–0.29; *p* = 0.50). We also analyzed CES-D scales as a continuous variable, but no significant association was found between continuous CES-D scale and TAC with coefficient of β = -0.003 (95% CI -0.013–0.006; *p* = 0.5) (Table 2). Furthermore, there was no evidence of sex-specific variation in this association.

**Table 2:** Association between CES-D and TAC Scores

	Univariate			Multivariable*		
	β	95% CI	*P*-value	β	95% CI	*P*-value
CES-D ≥ 16	-0.182	-0.445, 0.082	0.20	0.08	-0.13, 0.29	0.50
CES-D (continuous)	-0.020	-0.032, -0.008	0.001	-0.003	-0.013, 0.006	0.5

CES-D, Center for Epidemiologic Studies Depression Scale; CI, confidence interval

*Multivariable models were adjusted for age, sex, race/ethnicity, smoking, body mass index, total cholesterol, high-density lipoprotein cholesterol, lipid-lowering medication use, hypertensive medication use, diabetes, systolic blood pressure, health insurance status, education, and known antidepressant use

### Joint association of depressive symptoms and TAC scores with ASCVD events

During the follow-up period (median 10.35 years), a total of 230 ASCVD events (coronary heart disease death (*n* = 26), myocardial infarction (*n* = 80), resuscitated cardiac arrest (*n* = 3), and stroke (*n* = 121)) were recorded. In a Cox proportional hazards model adjusting for relevant covariates, both Exam 5 depressive symptoms and TAC were independently and positively associated with incident ASCVD events with HR = 1.63 (95% CI 1.16–2.29; *p* = 0.005) and HR = 1.22 (95% CI 1.13–1.33; *p* < 0.001), respectively (Table 3). No significant interaction was observed between the two predictors (interaction *p* = 0.551).

**Table 3:** Fully Adjusted Cox Model with ASCVD Event Endpoint

Characteristic	HR (95% CI)	*P*-value
Age (years)	1.03 [1.01, 1.05]	0.001
Male sex	1.75 [1.29,2.36]	< 0.001
Chinese American	0.649 [0.403, 1.04]	0.075
Black	0.920 [0.648, 1.31]	0.640
Hispanic/Latino	0.950 [0.643, 1.40]	0.794
Body mass index (kg/mm)	0.986 [0.958, 1.02]	0.342
Smoking (pack-years)	0.998 [0.993, 1.00]	0.601
High school	0.910 [0.619, 1.34]	0.630
Some college	0.519 [0.303, 0.888]	0.017
College and above	1.01 [0.633, 1.61]	0.966
Total cholesterol (mg/dl)	1.00 [1.00, 1.01]	0.016
HDL cholesterol (mg/dl)	0.993 [0.983, 1.00]	0.147
Diabetes	1.36 [0.990, 1.87]	0.057
Systolic blood pressure (mmHg)	1.01 [1.00, 1.02]	0.003
Lipid-lowering medications	1.11 [0.827, 1.48]	0.497
Hypertensive medications	0.730 [0.537, 0.993]	0.045
No health insurance	1.03 [0.597, 1.78]	0.910
Antidepressant	1.07 [0.685, 1.68]	0.760
Depressive symptom (CES-D ≥ 16)	1.63 [1.16, 2.29]	0.005
Log (TAC score + 1)	1.22 [1.13, 1.33]	< 0.001

ASCVD, atherosclerotic cardiovascular disease; CI, confidence interval; HDL, high-density lipoprotein; HR, hazard ratio; TAC, thoracic artery calcification

**Fig. 2:**
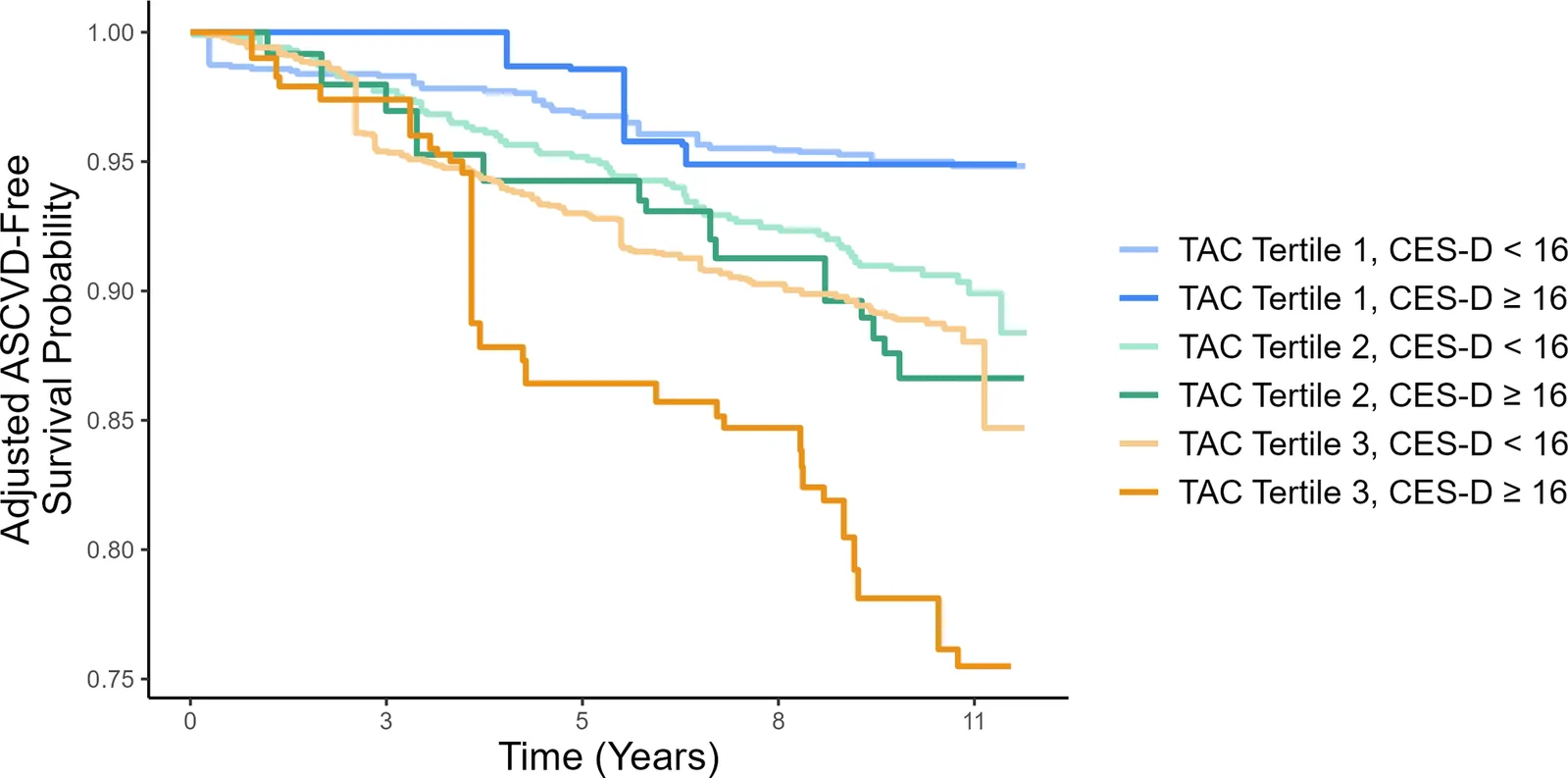
IPTW-weighted Kaplan-Meier ASCVD-free Survival Curves by TAC Tertile and Depressive Symptoms. Curves represent IPTW-weighted Kaplan-Meier survival estimates adjusting for relevant covariates. The lowest survival probability was observed among participants with depressive symptoms (CES-D ≥ 16) in the highest TAC tertile. ASCVD, atherosclerotic cardiovascular disease; CES-D, Center for Epidemiologic Studies Depression Scale; IPTW, inverse probability of treatment weighting; TAC, thoracic aortic calcification

To further evaluate the combined impact, we classified subjects into six groups based on the combination of TAC tertiles (T1, T2, T3) and CES-D status (CES-D ≥ 16 or < 16). IPTW-weighted Kaplan-Meier survival curves stratified by these groups are shown in Fig. 2. Participants with both depressive symptoms (CES-D ≥ 16) and severe TAC tertile (T3) exhibited the highest incidence of ASCVD events (log-rank, *p* < 0.001). Subgroup analysis revealed that the hazard of an ASCVD event did not differ by CES-D status within the first (HR = 1.107; 95% CI 0.423–2.894; *p* = 0.837) and the second TAC tertiles (HR = 1.5; 95% CI 0.833–2.698; *p* = 0.176). However, within the third TAC tertile (highest burden), participants with CES-D ≥ 16 had a significantly higher hazard of ASCVD compared to those with CES-D < 16 within the same tertile (HR = 1.897; 95% CI 1.203–2.990; *p* = 0.006) after adjusting for relevant covariates (Table 4). In a sensitivity analysis also adjusting for current alcohol use and moderate-to-vigorous physical activity, the associations of our primary predictors with ASCVD events remained robust (CES-D ≥ 16: HR 1.61, 95% CI 1.15–2.27, *p* = 0.006; and log(TAC + 1): HR 1.22, 95% CI 1.12–1.33, *p* < 0.001) (Supplemental Table 1).

**Table 4:** Hazard Ratios for ASCVD Comparing CES-D ≥ 16 vs. < 16 Within Each TAC Tertile

Tertile of TAC	HR of CES-D ≥ 16	95% CI	*P*-value
Tertile 1	1.107	0.423, 2.894	0.837
Tertile 2	1.500	0.833, 2.698	0.176
Tertile 3	1.897	1.203, 2.990	0.006

CES-D, Center for Epidemiologic Studies Depression Scale; CI, confidence interval; TAC, thoracic artery calcification

## Discussion

This study is the first to evaluate the direct association between depressive symptoms and atherosclerosis using TAC, as well as their joint prognostic value for ASCVD, in a large multiethnic cohort. We found no significant association between depressive symptoms and TAC, and no evidence of sex-specific differences in this relationship. However, depressive symptoms and TAC were each independently associated with incident ASCVD events, and in subgroup analysis, participants with both depressive symptoms and elevated TAC demonstrated the highest ASCVD risk. These findings highlight the potential clinical importance of incorporating depressive symptom assessment into cardiovascular risk stratification, particularly among individuals with evidence of advanced subclinical atherosclerosis.

### Depressive symptoms and atherosclerosis

In our analysis, we observed no significant direct association between depressive symptoms based on the CES-D scale and TAC. Several previous studies have examined the relationship between depressive symptoms and aortic calcification from the aortic arch to the iliac bifurcation. For example, Agatisa et al. analyzed 210 healthy, middle-aged women from the SWAN Heart Study and found that a history of recurrent major depression was associated with a more than three-fold increase in the risk of aortic calcification (OR 3.39; 95% CI 1.34–8.63) [10]. Similarly, Matthews et al. examined 155 women in the Healthy Women Study, reporting that higher CES-D scores significantly predicted a greater calcification burden (OR 1.57; 95% CI 1.04–2.36) [11]. The most plausible explanation for the discrepancy between prior studies and our findings is the difference in the age of the study populations. The mean age of participants in our cohort was 68 years, substantially older than those in earlier studies, which primarily included younger or middle-aged individuals. In older populations, aortic calcification likely reflects long-standing cumulative atherosclerotic exposure, and the incremental contribution of depressive symptoms to calcified plaque burden may be attenuated or obscured by age-related vascular changes and traditional cardiovascular risk factors. Further studies across diverse populations are needed to better elucidate the relationship between depressive symptoms and atherosclerosis.

### Joint association with ASCVD risk

Our study demonstrated that depressive symptoms were independently associated with ASCVD despite the absence of a significant association between depressive symptoms and TAC. These findings suggest that the primary mechanism linking depression to ASCVD may not be mediated through atherosclerotic role of depression in advanced disease. However, the coexistence of depression and elevated TAC was associated with a higher risk of ASCVD events, which may reflect increased vulnerability in this subgroup. The interplay between depression and ASCVD, as well as the worse prognosis observed when they coexist, may be explained by both behavioral and biological mechanisms [17]. Depression adversely affects adherence to medication and healthy behaviors and is associated with an increased risk of ASCVD, particularly among high-risk individuals [18]. In addition, several biological pathways, including autonomic dysregulation, inflammation, and prothrombotic states, are disrupted in both depression and ischemic heart disease. Autonomic dysregulation associated with depression (increased sympathetic and decreased parasympathetic activity) reduces heart rate variability, leading to higher ASCVD risks and mortality [19, 20]. Individuals with depression exhibit chronic low-grade inflammation and increased platelet reactivity, both of which are established predictors of thrombotic events [21, 22]. Through these pathophysiological mechanisms, depressive symptoms may contribute to an increased risk of ASCVD events, and individuals with both depressive symptoms and subclinical atherosclerosis may experience higher risk due to the coexistence of these factors.

### Clinical implications

Our findings highlight the potential value of incorporating psychological factors into cardiovascular risk stratification, particularly in primary prevention settings. Depression screening is not routinely performed in cardiovascular care but may be especially important among individuals with severe atherosclerosis or multiple cardiovascular risk factors. Cardiologists and primary care providers play a critical role in initiating depression screening and collaborating within a multidisciplinary care model that includes mental health specialists. Early identification of depression enables timely intervention, which may improve both psychological and cardiovascular outcomes. Moreover, we did not observe an association between antidepressant use and increased ASCVD risk, supporting the safety of pharmacologic treatment for depression when clinically indicated. In addition, nonpharmacologic interventions, including promotion of physical activity and improved adherence to cardiovascular medications, remain essential components of comprehensive care.

### Limitations

There are several limitations associated with this study. First, this analysis was based on data from MESA Exam 5, which took place approximately 10 years after the initial enrollment. Consequently, the average age of participants in this analysis was 68 years, which may have introduced a degree of survivor bias and makes the generalization of these findings to younger populations difficult. Second, while we controlled for antidepressant use, we did not account for other potentially relevant medications, such as anticoagulants, which could influence ASCVD outcomes. Furthermore, as this is still an early study to investigate the direct association between depressive symptoms and TAC using non-contrast chest CT, these results should not be interpreted as definitive answers. Future research using diverse, younger cohorts is necessary to confirm these associations and to evaluate whether the joint prognostic value of depression and TAC remains consistent across various stages of the lifespan. Additional studies should also consider a broader range of pharmacological covariates to isolate the impact of psychological distress on vascular health.

## Conclusion

In this study, no direct association was observed between depressive symptoms and TAC, nor was there evidence of sex-specific variation in this relationship. However, depressive symptoms and higher TAC scores were each independently associated with an increased risk of future ASCVD events. Higher ASCVD event risk was observed among individuals with both depressive symptoms and elevated TAC, reflecting the coexistence of multiple risk factors. These findings suggest that evaluating depressive symptoms may provide additional context for cardiovascular risk stratification, particularly among individuals with evidence of substantial subclinical atherosclerotic burden.

## Supplementary Information

Below is the link to the electronic supplementary material.

Supplementary Material 1

## Data Availability

No datasets were generated or analysed during the current study.
