# Supplementary material for: Association between depressive symptoms and thoracic aortic calcification, and their independent and joint prognostic value for incident atherosclerotic cardiovascular disease in the MESA cohort
Source: Int J Cardiovasc Imaging. 2026 Aug 19;42(10):1973–81. doi: 10.1007/s10554-026-03771-0 (PMC13645938; doi:10.1007/s10554-026-03771-0)
Supplement: Supplementary file 1 [file 10554_2026_3771_MOESM1_ESM.docx]

**Supplementary Table 1.** Sensitivity Analysis Adjusting for Behavioral Covariates

| Characteristic |  |  | HR (95% CI) | P-value |  |
| --- | --- | --- | --- | --- | --- |
| CES-D (Exam 5) ≥ 16 |  |  | 1.61 [1.15, 2.27] | 0.006 |  |
| Log (TAC+1) |  |  | 1.22 [1.12, 1.33] | < 0.001 |  |
| Current Alcohol Use |  |  | 0.880 [0.654, 1.18] | 0.400 |  |
| Moderate and Vigorous Physical Activity Total (MET-hours/week) |  |  | 0.999 [0.998, 1.00] | 0.456 |  |
| Race/Ethnicity |  |  |  |  |  |
| White |  |  | — |  |  |
| Black |  |  | 0.910 [0.640, 1.29] | 0.601 |  |
| Hispanic/Latino |  |  | 0.931 [0.629, 1.38] | 0.720 |  |
| Chinese |  |  | 0.611 [0.375, 0.996] | 0.048 |  |
| Age |  |  | 1.03 [1.01, 1.05] | 0.002 |  |
| Sex (Male) |  |  | 1.81 [1.33, 2.46] | < 0.001 |  |
| BMI (kg/m²) |  |  | 0.985 [0.957, 1.01] | 0.323 |  |
| Systolic Blood Pressure (mm Hg) |  |  | 1.01 [1.00, 1.02] | 0.003 |  |
| Pack Years Smoking |  |  | 0.999 [0.993, 1.00] | 0.633 |  |
| Diabetes |  |  | 1.35 [0.983, 1.86] | 0.064 |  |
| Total Cholesterol (mg/dl) |  |  | 1.01 [1.00, 1.01] | 0.014 |  |
| HDL Cholesterol (mg/dl) |  |  | 0.994 [0.984, 1.00] | 0.191 |  |
| Takes Hypertension Medication |  |  | 0.736 [0.540, 1.00] | 0.051 |  |
| Takes Lipid-Lowering Medication |  |  | 1.12 [0.835, 1.49] | 0.456 |  |
| No Health Insurance |  |  | 1.02 [0.588, 1.76] | 0.951 |  |
| Education |  |  |  |  |  |
| Less Than High School |  |  | — |  |  |
| High School Graduate |  |  | 0.932 [0.632, 1.37] | 0.721 |  |
| College |  |  | 0.539 [0.313, 0.926] | 0.025 |  |
| Graduate School |  |  | 1.05 [0.657, 1.69] | 0.825 |  |
| Known Antidepressant Use |  |  | 1.05 [0.672, 1.65] | 0.824 |  |

CES-D, Center for Epidemiologic Studies Depression Scale; CI, confidence interval; HDL, high-density lipoprotein; HR, hazard ratio; MET, metabolic equivalent of task; TAC, thoracic artery calcification.
